# Differential abundance of IgG antibodies against the spike protein of SARS-CoV-2 and seasonal coronaviruses in patients with fatal COVID-19

**DOI:** 10.1186/s12985-023-02050-x

**Published:** 2023-05-03

**Authors:** Wouter L. Smit, Sophie van Tol, Lenneke E. M. Haas, Gijs J. M. Limonard, Ailko Bossink, Chantal Reusken, Michiel Heron, Steven F. T. Thijsen

**Affiliations:** 1 Present Address: Department of Medical Microbiology and Immunology, Diakonessenhuis Utrecht, Bosboomstraat 1, 3582 KE Utrecht, The Netherlands; 2 Department of Medical Microbiology, University Medical Center Utrecht, Heidelberglaan 100, 3584 CX Utrecht, The Netherlands; 3 Centre for Infectious Disease Control, WHO Reference Laboratory for COVID-19, National Institute for Public Health and the Environment (RIVM), Bilthoven, The Netherlands; 4 Department of Intensive Care, Diakonessenhuis Utrecht, Utrecht, The Netherlands; 5 Department of Pulmonary Diseases, Diakonessenhuis Utrecht, Utrecht, The Netherlands

## Abstract

Infection with the novel pandemic SARS-CoV-2 virus has been shown to elicit a cross-reactive immune response that could lead to a back-boost of memory recall to previously encountered seasonal (endemic) coronaviruses (eCoVs). Whether this response is associated with a fatal clinical outcome in patients with severe COVID-19 remains unclear. In a cohort of hospitalized patients, we have previously shown that heterologous immune responses to eCoVs can be detected in severe COVID-19. Here, we report that COVID-19 patients with fatal disease have decreased SARS-CoV-2 neutralizing antibody titers at hospital admission, which correlated with lower SARS-CoV-2 spike-specific IgG and was paralleled by a relative abundance of IgG against spike protein of eCoVs of the genus Betacoronavirus. Additional research is needed to assess if eCoV-specific back-boosted IgG is a bystander phenomenon in severe COVID-19, or a factor that influences the development of an efficient anti-viral immune response.

**Supplementary Information:**

The online version contains supplementary material available at 10.1186/s12985-023-02050-x.

## Introduction

Severe acute respiratory syndrome coronavirus 2 (SARS-CoV-2) causes a clinical spectrum ranging from asymptomatic infections to severe COVID-19 pneumonia that requires hospitalization and admission to the pulmonary or intensive care unit (ICU). A proportion of critically ill patients, predominated by elderly or patients with pre-existing comorbidities [1], exhibit a dysregulated immune response characterized by impaired type I interferon activity [2, 3], delayed neutralizing antibody production [4], dysregulated cellular immunity [5, 6], and excessive cytokine release (cytokine storm) which can be blocked in order to mitigate severe disease [7]. This indicates a maladapted immune response that contributes to a fatal disease course of which the mechanism is poorly understood.

A phenomenon that may potentially alter the disease course of COVID-19 is a back-boost of humoral immunity due to memory recall resulting from previous exposure to endemic/seasonal human coronaviruses (HCoVs; HCoV-OC43, HCoV-HKU1, HCoV-229E and HCoV-NL63), from here on referred to as eCoVs. Recent evidence suggests there is extensive cross-reactivity of antibodies directed at the relatively conserved S2-subunit of the spike protein of these coronaviruses, although this does not appear to be cross-protective in COVID-19 [8, 9]. Furthermore, a recent study found evidence that memory B cell clones reactive to the OC43 were enriched due to a back-boost effect, in patients with severe COVID-19, showing poorly SARS-CoV-2 neutralizing capacity in vitro [10]. In our cohort, we have previously reported heterologous immunity suggestive of a similar back-boost effect directed against the spike protein in severe COVID-19, with strong correlations of anti-spike IgG against SARS-CoV-2 and eCoVs of the genus Betacoronavirus (OC43 and HKU1) in severely ill patients [11]. Following up on this work, we here assess neutralizing immunity using VNT assays and evaluate the relationship with anti-spike IgG titers against SARS-CoV-2 and the eCoVs in our cohort of severe COVID-19 patients. We focus on the subgroup of deceased patients in order to identify potential immune correlates of a fatal clinical outcome.

## Results

### Patients with a fatal disease outcome have lower neutralizing antibody titers at hospital admission that correlate with IgG responses against Betacoronavirus spike protein

55 patients admitted to the intensive care unit (ICU) or the pulmonary ward during the first or second wave of SARS-CoV-2 infections in 2020 in the Netherlands with a severe form of COVID-19 pneumonia were enrolled as part of the SARS-CoV-2 immune response (SIR) study and used for further analysis. Sera were collected upon confirmation of SARS-CoV-2 infection with a validated in-house PCR test. Concurrently, a group of 61 healthy volunteers was included, of which 27 had a history of asymptomatic or mild COVID-19 based on serostatus and a reported positive PCR at the time of infection in a majority of cases [12]. Patients with severe disease were divided into individuals who recovered during follow-up (termed non-fatal disease) and individuals who deceased during hospitalization or up to one week after discharge (termed fatal disease). Baseline characteristics of each of these cohort groups are shown in Additional file 1: Table S1. As expected based on known risk factors of COVID-19, patients with a fatal disease course were significantly older and more frequently had age-related comorbidies including heart disease, hypertension, respiratory disease, and cancer of any type (Additional file 1: Table S1). Importantly, the time between self-reported symptom onset and sampling did not differ between both groups.

Next, we assessed IgG responses against the spike protein of each of the eCoVs as well as SARS-CoV-2 (Wuhan-Hu-1 strain) using a protein array that allows for parallel and quantitative assessment of antigen-specific IgG, as previously reported [11, 13]. We also measured virus neutralization titers (VNT) against SARS-CoV-2 in the same samples to correlate SARS-CoV-2 and eCoV-specific circulating IgG titers to the virus neutralization capacity. Epitopes of the recombinant antigens used spanned the full spike protein, with in vitro modifications shown in supplementary Additional file 1: Fig. S1. Additionally, we made use of a S1-subunit specific antigens specific to each of the coronaviruses.

**Fig. 1:**
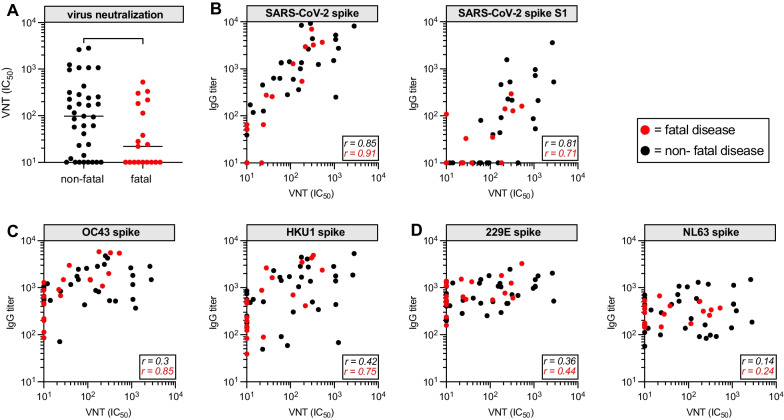
Correlation to circulating anti-spike IgG antibodies of coronaviruses and SARS-CoV-2 virus neutralization. **A** Virus neuralization titers in fatal and non-fatal COVID-19 patients. Mann–Whitney U test; *P < 0.05. **B** Correlation between virus neutralization titer and anti-spike IgG of SARS-CoV-2 and the various eCoVs are shown with Spearman rank coefficients shown in the adjacent brackets. Red and black dots respectively represent the fatal and non-fatal patients. **C** Same as in (**B**) for the eCoVs of the genus Betacoronavirus. **D** Same is in (**B**) for the eCoVs of the genus Alphacoronavirus

First, we analyzed virus neutralization in non-fatal and fatal COVID-19 patients at hospital admission. VNT, expressed as the log 10 of the IC_50_ titer, was significantly lower in the fatal disease group (*P* = 0.04) (Fig. 1A). Because patients were sampled at day of hospital admission, time between symptom onset and sampling varied within both groups, and likely therefor also duration of infection. To account for this, we rearranged the data according to days after (patient self-reported) symptom onset. Titers in the non-fatal disease group were increasing over time, which shows mounting of an immune response (Additional file 1: Fig. S2). However, titers in the fatal disease group were already lower after 1 week and eventually reached lower levels, although comparative differences between groups per time-point were not statistically significant due to the limited number of data-points available to this analysis. Although fatal patients were significantly older on average compared to non-fatal patients, a very weak inverse correlation with VNT (Additional file 1: Fig. S3A) and absent correlation with antibody titers (Additional file 1: Fig. S3B) was found, when samples were sorted on age. Furthermore, SARS-CoV-2 nucleacapsid-specific responses were not significantly lower, indicating that fatal patients were still capable of mounting a (quantitative) immune response (Additional file 1: Fig. S3C).

To identify immune correlates of a neutralizing response, we correlated VNT to anti-spike IgG of each of the coronaviruses. As expected [14], IgG against SARS-CoV-2 spike correlated strongly with the VNT in both the fatal and non-fatal group (Spearman r = 0.91 and 0.85, respectively) (Fig. 1B). Epitopes on S1 are likely to be important for neutralization, given that the S1-subunit specific IgG titer also correlated strongly with VNT in both the fatal and non-fatal group (Spearman r = 0.71 and 0.81, respectively). Notably, antibodies against the eCoVs of the genus Betacoronavirus (OC43 and HKU1) also showed a positive correlation with VNT (Fig. 1C), whilst no correlations were found for the Alphacoronaviruses (229E and NL63) (Fig. 1D). The strongest correlations with OC43 and HKU1 titers were seen in the fatal disease group (Spearman r = 0.85 and 0.75 for OC43 and HKU1, respectively). In contrast, no correlation with any of the eCoVs was observed against the S1-subunit of spike, which implies that immunogenicity to the S2-subunit underpins the observed correlations (Additional file 1: Fig. S4).

**Fig. 2:**
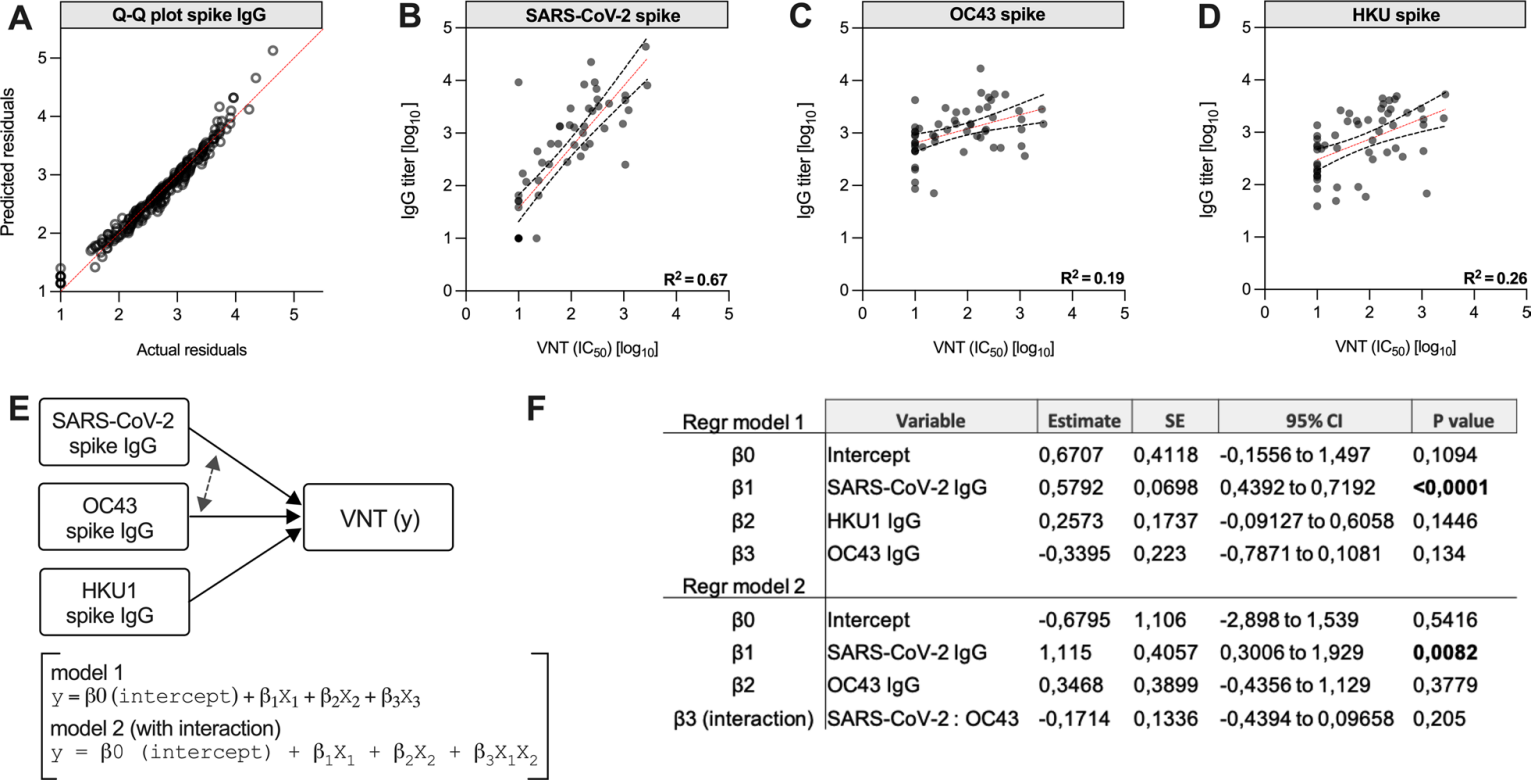
Simple and multiple linear regression analysis of Betacoronavirus spike IgG titers and VNT. **A** Q-Q plot of residuals of log-transformed titer data to assess normality. Data passed the normality test (D’agostino-Pearson Omnibus test, *P* = 0.01). **B** Simple linear regression to estimate the relationship between SARS-CoV-2 spike IgG and the virus neutralization titer (VNT). **C** same as (**B**) but for OC-43 spike IgG **D** same as (**B**) but for HKU1 spike IgG. **E** Main effects model the of Betacoronavirus (SARS-CoV-2, OC43, HKU1) spike IgG titers and SARS-CoV-2 virus neutralization (VNT). Models with and without potential interaction were analyzed (only interactions between eCoV-spike and SARS-CoV-2 on VNT). Regression formula for models with and without assumed interactions are shown below. **F** Tabular results of the statistics: SARS-CoV-2 IgG titer was a strong predictor of VNT, and no significant effect of the other variables on VNT could be shown. Significant values are denoted in bold in the table

Next, we aimed to model the relationship between the Betacoronavirus-specific IgG titers and VNT to assess if change in neutralization can be predicted by the presence of each of these antibody titers, and if interacting effects can be identified in a model. The titer and VNT data were log-transformed into an approximately normal distribution (D’agostino Pearson test passed, *P* = 0.01, Q-Q plot of residuals shown in Fig. 2A). A simple linear regression model with best fit using VNT as the response (dependent) variable was calculated for each antibody titer (SARS-CoV-2, OC43, HKU1). As shown, the SARS-CoV-2 titer explained most the variation in VNT (Fig. 2B), and only weak coeffients were found for OC43 or HKU1 (Fig. 2C,D). We also performed multiple linear regression analyses with and without interactions, since the theory of antigenic imprinting assumes that back-boosted eCoV-specific antibodies can interfere with SARS-CoV-2 antibodies to affect the neutralization capacity (Fig. 2E). We thus calculated main effects of Betacoronavirus spike IgG titers (explanatory variables) on the VNT (response variable). The SARS-CoV-2 titer alone strongly predicted the VNT and no significant interaction effects of OC43/HKU IgG could be observed in the regression model (Fig. 2F, Additional file 1: Tables S2-S4).

**Fig. 3:**
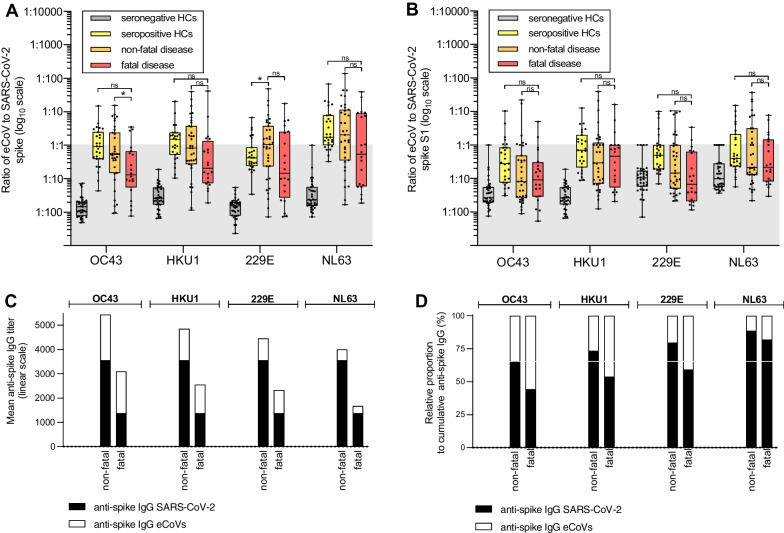
Ratio of SARS-CoV-2 anti-spike IgG to eCoV anti-spike IgG and relative proportions in severe COVID-19 patients and healthy controls. **A** Ratio of eCoV anti-spike IgG response to SARS-CoV-2 response. The gray division in the figure indicates the point at which the SARS-CoV-2: eCoV ratio response is below 1. Disease groups and healthy controls (HC) are shown separately in differently colored boxes, which represent the median values and interquartile range with whiskers showing minima and maxima. Statistical differences were calculated with a one-way ANOVA test. **B** Similar ratios as in (**A**) but responses against the S1 subunit of spike. **C** Anti-spike IgG levels are shown with the length of the bars reflecting the mean titer in the non-fatal and fatal cohort. **D** Relative contributions (%) to the cumulative (SARS-CoV-2 + eCoV) anti-spike response, calculated from (**C**) to show relative abundances

Recently a similar study assessed comparisons of immune responses against eCoVs and SARS-CoV-2 in a cohort of ICU patients, comprising a subset of individuals with a fatal clinical outcome [15]. The authors also demonstrated a positive correlation between IgG titers directed against the spike protein of SARS-CoV-2, OC43 and HKU1, predominantly in patients with fatal disease. Correlating responses were driven by reactivity to epitopes in the more conserved S2-subunit. Furthermore, enrichment or relative abundance of the humoral immune response to OC43 and HKU1 was noted in patients with fatal disease, when analyzed as ratio to the SARS-CoV-2-specific response [15]. We performed a similar analysis in our disease cohort. To assess  “background exposure” to the circulating eCoVs, we included a subsets of seropositive and seronegative healthy controls (HCs). These HCs were younger (mean age of 41 yrs) and had not yet received a SARS-CoV-2 vaccine yet. Next, the proportion of eCoV-specific IgG responses to spike was expressed as the titer fold ratio of SARS-CoV-2 IgG over eCoV IgG (SARS-CoV-2: eCoV) within each group separately (Fig. 3A). The ratio of seropositive HCs was similar to patients for each of the eCoVs, with exception of the response to 229E where HCs showed a significantly lower ratio compared to non-fatal patients. This may reflect a different history of exposure between the younger HCs and the older patients. Notably, lower SARS-CoV-2: eCoV ratios, indicative of a relatively abundance of anti-spike antibodies reactive to the eCoVs, were seen in fatal compared to non-fatal patients, although this was only statistically significant for OC43 (*P* = 0.04, one-way ANOVA). No differences were observed when assessing S1-subunit specific responses (Fig. 3B). When comparing the non-fatal to the fatal disease group, it should be emphasized that the lower SARS-CoV-2: eCoV ratio is the results of a relatively lower SARS-CoV-2- specific titers in the latter, instead of higher eCoV specific titers (Fig. 3C). As such, the cumulative anti-spike response was found to be enriched for eCoV-specific IgG, with a majority reacting to OC43 (Fig. 3D).

## Discussion

In this descriptive study we shown that cross-reactive humoral responses directed at the spike protein of OC43 and HKU1 correlate with SARS-CoV-2 virus neutralization in severe COVID-19, predominantly in patients with fatal disease. No relationship was observed with S1-subunit-specific responses, indicating involvement of the more conserved epitopes in the S2-subunit. Virus neutralization was significantly reduced in patients with fatal disease, but no evidence of interfering OC43 or HKU1-specific antibodies on the VNT could be found using regression models. Finally, when looking at cumulative IgG titers, OC43-specific responses predominate in fatal disease. No causation can be inferred from this data, and additional research is needed to explore whether back-boostinged could interfere with mounting an efficient antiviral immune response to SARS-CoV-2, or acts as a bystander.

Exposure to epitopes from antigen of a foreign pathogen that closely resemble epitopes of a previously encountered pathogen, either due to antigenic drift or vaccine-derived, are well-described phenomena in virology, in particular in relation to influenzaviruses [16]. The coronavirus family also shares a substantial degree of homogeneity in epitopes embedded in the S2-subunit of spike, producing cross-reactive antibodies to circulating eCoVs in COVID-19 [17]. Whether this is a bystander effect, or a factor that interferes with development of the antiviral immune response to SARS-CoV-2 is an important open question.

Several recently published studies have proposed that preexisting immunity to eCoVs could impede or delay mounting of humoral response after a first encounter with the novel coronavirus [10, 15, 18, 19]. Such antigenic imprinting mechanism, often referred to as antigenic sin [20], was primarily based on correlating responses (IgG and IgA) to the spike protein (S2-domain) that appear to be poorly neutralizing [15, 18], although only a few studies have sought to probe deeper into the mechanism. For instance by showing that affinity purified OC-43-specific IgG [19], or sorted OC-43 spike reactive B cell clones [10], show poor neutralization capacity. This study and our previous work [11] reconfirms presence of cross-reactive responses to genus Betacoronavirus eCoVs and SARS-CoV-2 spike that are likely the result of back-boosting in severe COVID-19. We could not show an effect of back-boosted IgG on virus neutralization (no significant interaction on the dynamics of VNT in our cohort using regression modeling, and also no invert correlation), although antigenic imprinting cannot be excluded due to a need for additional functional experiments and limitations inherent to our study design.

First of all, the group of fatal patients was limited in number which did not allow for an age-matching; this potentially renders correlations prone to multicollinearity, although the calculated Variance Inflation Factors (VIF) were below 3 in the linear regression analyses, which argues against this. Second of all, we could not measure S2-subunit-specific responses and had to infer the involvement of S2 based on the differential response between the full length spike and S1. Also, we did not have recombinant eCoV-specific nucleocapsid to our disposal. Third of all, and most importantly, our study did not include serial samples per individual from day of symptom onset to weeks after onset of the infection, to assess intra-individual immune kinetics. Such study design is logistically challenging and these cohorts are rare, but this study type is crucial to address the hypothesis of antigenic imprinting. Future (retrospective) studies are thus needed that assess the impact of back-boosted antibodies on derailed antiviral immunity in severely ill patients (impaired neutralization, facilitation of viral entry) during the initial wave of SARS-CoV-2 infections. Of note, the role of vaccinations must be taken into account as this might “reset” the memory B cell compartment in a way that removes the potential constrains of immune imprinting [22].

## Methods

### Study cohort

Patients who were admitted to the Diakonessenhuis hospital from May 2020 with COVID-19 disease were enrolled in the SIR study once SARS-CoV-2 infection was confirmed with PCR on nasal mucosa or bronchial excrete. Patients were either included from the pulmonary ward or directly from the ICU, and no exclusion criteria were used. Medical history was retrieved from the medical records. Healthy controls consisted of hospital personnel, without a medical history of a condition that could be expected to influence their immune status or the natural history of COVID-19 disease, who voluntarily participated in the study. All healthy controls were vaccine-naïve, individuals who had already been vaccinated were excluded.

### Protein expression

Secreted recombinant protein were produced in-house by transfection of plasmid DNA in mammalian HEK293F suspension cells as previously described [23]. The transfected pPPI4 plasmids contained a sequence encoding for the corresponding antigen, followed by a HIS-tag. The following antigens were used: spike protein trimer and S1 subunit of SARS-CoV-2 (GenBank QHD43416.1), HCoV-229E (GenBank JX503061.1), HCoV-HKU1 (GenBank ADN03339.1), HCoV-OC43 (GenBank AIX10763), HCoV-NL63 (GenBank ABE97130.1) and MERS-CoV (GenBank KJ650297.1). In case of spike trimer proteins, the HIS-tag was preceded by a trimerization motif. Here, a prefusion-stabilized S protein ectodomain of SARS-CoV-2 and eCoVs with a T4 trimerization domain and hexahistidine (His) tag was designed as previously described [24]. Secreted recombinant protein was purified from the cell suspension by gravity flow chromatography using Ni–Nta beads. For spike trimer proteins, monomers and dimers were excluded from the mixture using size exclusion chromatography. For spike S1, dimerization and complex formation was excluded. Preparation of human coronavirus protein micro-array was based on previously described methodology [13].

### Protein microarray

Sera were tested in 8 threefold dilutions starting at 1:10, diluted in Blotto buffer containing 0.1% Surfact-Amps20 (ThermoFisher). Goat anti-human IgG, F(ab’)2 fragment specific, Alexa Fluor 647-conjugated (Jackson Immuno Research, West Grove, USA) was used in a 1:1000 dilution in Blotto buffer containing 0.1% Surfact-Amps20. A pool of SARS-CoV-2 positive control samples with known titers to eCoVs and SARS-CoV-2, was included in every run to assess low inter-assay variation. For analysis, ScanArray Express software version 4.0.0.0004 (PerkinElmer, Waltham, USA) was used to quantify fluorescent signals. The mean of the median spot fluorescence of duplo measurements was plotted into dose–response curves per antigen for each serum using R studio v4.0.0, package “DRC” version 2.3–7 (R studio, Boston, USA). A representative theoretical antibody titer (EC50) was chosen at the 50% response on the dose–response curve, analogous to the median infectious dose (ID50) in the dose–response theory. If the median fluorescence measurements of the used serum dilutions were outside the linear range of the sigmoidal dose response curves, no titer could be calculated.

### Virus neutralization assay

SARS-CoV-2 neutralization assays were performed as described previously by Rijkers et al^25^. (2020) In brief, serum samples were heat-inactivated at 56◦C for 30 min. Duplicate serial twofold dilutions starting at 1:10 were mixed with 100 TCID50 SARS-CoV-2 [strain hCoV-19/Netherlands/ZuidHolland_10004/2020, D614G (WT)] in a 96-wells format and incubated for 1 h at 35◦Cand 5% CO2. Subsequently, VeroE6 cells (1.8 × 104 cells/well) were added.and then incubated for 3 days at 35◦C and 5% CO2. The plates were assessed microscopically by scoring 50% protection for cytopathic effect and the titer (VNT50) was defined as the reciprocal value of the serum dilution showing 50% virus neutralization. Titers ≥ 10 were considered seropositive. All laboratory procedures using live SARS-CoV-2 were performed in a biosafety level 3 facility.

### Statistical analysis

Statistical analyses were performed using GraphPad Prism version 9.5.1. Correlations were calculated with Spearman's rank correlation coefficient on the untransformed data. Log-transformation on protein array-based IgG titers (spot fluorescence) and VNT data was carried out to normalize the distribution, followed by regression analysis (simple linear regression and multiple linear regression). Interpretation of the coefficient of determination were as follows: a coefficient of 0–0.25 indicates little to no variance explained, 0.25–0.5 indicates small amount of variance explained, 0.5–0.75 indicates good amount of variance explained, 0.75 -1 indicates significant amount of variance explained. For multiple regression modeling, intercept and mean effects with or without the presence of interactions was selected in GraphPad Prism (under tab multiple variable analyses), producing a tabular statistics sheet that is included in the Additional file 1: Tables. When comparing (unpaired) continuous values between groups, we used Mann–Whitney U test or one-way ANOVA when assessing two or more groups, respectively. Levels of significance: *P ≤ 0.05; **P ≤ 0.001; ***P ≤ 0.0001; ns = not significant.

## Supplementary Information

**Additional file 1. Figure S1.** Schematic linear representation of the antigens used for the protein array platform. The top bar is a linear representation of the design of recombinant stabilized prefusion SARS-CoV-2 Spike ectodomain with the signal peptide shown in blue and the S1 (red) and S2 (yellow), where the furin cleavage site is replaced with a glycine linker (GGGG), two proline mutations are introduced (K986P and V987P), and a trimerization domain (cyan) preceded by a linker (GSGG) is attached. The four recombinant eCoV Spike proteins are shown below, in which the natural furin cleavage site is mutated in the case of HKU1 and OC43. **Figure S2.** VNT in non-fatal and fatal COVID-19 patients based on time after symptom onset. Median virus neutralization titers are shown in non-fatal and fatal COVID-19 patients stratified per time between symptom onset and sampling. **Figure S3.** Analysis of VNT and anti-spike antibody titers in patients sorted by age. **A** Correlation analysis (Spearman r) between VNT and age. **B** Correlation analysis (Spearman r) between antibody titers against SARS-CoV-2, OC43 and HKU1 spike and age. **C** SARS-CoV-2 nucleocapsid-specific IgG titer in patients with fatal and non-fatal disease (Mann–Whitney U test). **Figure S4.** Correlations between virus neutralization titer and IgG against the spike S1-subunit of eCoVs. Correlations are shown between the SARS-CoV-2 virus neutralization titer and the IgG titer against the S1 subunit of the eCoVs, with Spearman rank (r) values indicated in the brackets within each plot. In red and black are the samples from patients with fatal and non-fatal disease respectively. **Table S1.** Demografic characteristics of the patient cohort. Fatal COVID-19 cases comprised of patients with severe pneumonia who deceased during their stay in the hospital or up to 1 week after discharge. Significant differences (p < 0.05) are indicated in the right column (statistical test for multiple comparisons: ordinary one-way ANOVA). **Table S2.** Tabular statistics of multiple linear regression analysis without interaction. **Table S3.** Tabular statistics of multiple linear regression analysis with interaction OC43: SARS-CoV2. **Table S4.** Tabular statistics of multiple linear regression analysis with interaction HKU1: SARS-CoV2.

## Data Availability

The datasets generated and/or analyzed during the current study are are available on request from the corresponding author, WLS.
