# Supplementary material for: Differential abundance of IgG antibodies against the spike protein of SARS-CoV-2 and seasonal coronaviruses in patients with fatal COVID-19
Source: Virol J. 2023 May 3;20:85. doi: 10.1186/s12985-023-02050-x (PMC10156070; doi:10.1186/s12985-023-02050-x)
Supplement: Supplementary file 1 — Additional file 1. Figure S1. Schematic linear representation of the antigens used for the protein array platform. The top bar is a linear representation of the design of recombinant stabilized prefusion SARS-CoV-2 Spike ectodomain with the signal peptide shown in blue and the S1 (red) and S2 (yellow), where the furin cleavage site is replaced with a glycine linker (GGGG), two proline mutations are introduced (K986P and V987P), and a trimerization domain (cyan) preceded by a linker (GSGG) is attached. The four recombinant eCoV Spike proteins are shown below, in which the natural furin cleavage site is mutated in the case of HKU1 and OC43. Figure S2. VNT in non-fatal and fatal COVID-19 patients based on time after symptom onset. Median virus neutralization titers are shown in non-fatal and fatal COVID-19 patients stratified per time between symptom onset and sampling. Figure S3. Analysis of VNT and anti-spike antibody titers in patients sorted by age. A Correlation analysis (Spearman r) between VNT and age. B Correlation analysis (Spearman r) between antibody titers against SARS-CoV-2, OC43 and HKU1 spike and age. C SARS-CoV-2 nucleocapsid-specific IgG titer in patients with fatal and non-fatal disease (Mann–Whitney U test). Figure S4. Correlations between virus neutralization titer and IgG against the spike S1-subunit of eCoVs. Correlations are shown between the SARS-CoV-2 virus neutralization titer and the IgG titer against the S1 subunit of the eCoVs, with Spearman rank (r) values indicated in the brackets within each plot. In red and black are the samples from patients with fatal and non-fatal disease respectively. Table S1. Demografic characteristics of the patient cohort. Fatal COVID-19 cases comprised of patients with severe pneumonia who deceased during their stay in the hospital or up to 1 week after discharge. Significant differences (p < 0.05) are indicated in the right column (statistical test for multiple comparisons: ordinary one-way ANOVA). Table S2. T [file 12985_2023_2050_MOESM1_ESM.docx]

**Supplementary Figures**

***Figure S1. Schematic linear representation of the antigens used for the protein array platform***

*The top bar is a linear representation of the design of recombinant stabilized prefusion SARS-CoV-2 Spike ectodomain with the signal peptide shown in blue and the S1 (red) and S2 (yellow), where the furin cleavage site is replaced with a glycine linker (GGGG), two proline mutations are introduced (K986P and V987P), and a trimerization domain (cyan) preceded by a linker (GSGG) is attached. The four recombinant eCoV Spike proteins are shown below, in which the natural furin cleavage site is mutated in the case of HKU1 and OC43.*

**

***Figure S2. VNT in non-fatal and fatal COVID-19 patients based on time after symptom onset.***

*Median virus neutralization titers are shown in non-fatal and fatal COVID-19 patients stratified per time between symptom onset and sampling.* ***Figure S3. Analysis of VNT and anti-spike antibody titers in patients sorted by age.***

*A) Correlation analysis (Spearman r) between VNT and age. B) Correlation analysis (Spearman r) between antibody titers against SARS-CoV-2, OC43 and HKU1 spike and age. C) SARS-CoV-2 nucleocapsid-specific IgG titer in patients with fatal and non-fatal disease (Mann–Whitney U test).*

**

***Figure S4. Correlations between virus neutralization titer and IgG against the spike S1-subunit of eCoVs.***

*Correlations are shown between the SARS-CoV-2 virus neutralization titer and the IgG titer against the S1 subunit of the eCoVs, with Spearman rank (r) values indicated in the brackets within each plot. In red and black are the samples from patients with fatal and non-fatal disease respectively.*

**Supplementary Tables**

**

***Table S1. Demografic characteristics of the patient cohort.***

*Fatal COVID-19 cases comprised of patients with severe pneumonia who deceased during their stay in the hospital or up to 1 week after discharge. Significant differences (p < 0.05) are indicated in the right column (statistical test for multiple comparisons: ordinary one-way ANOVA).*

***
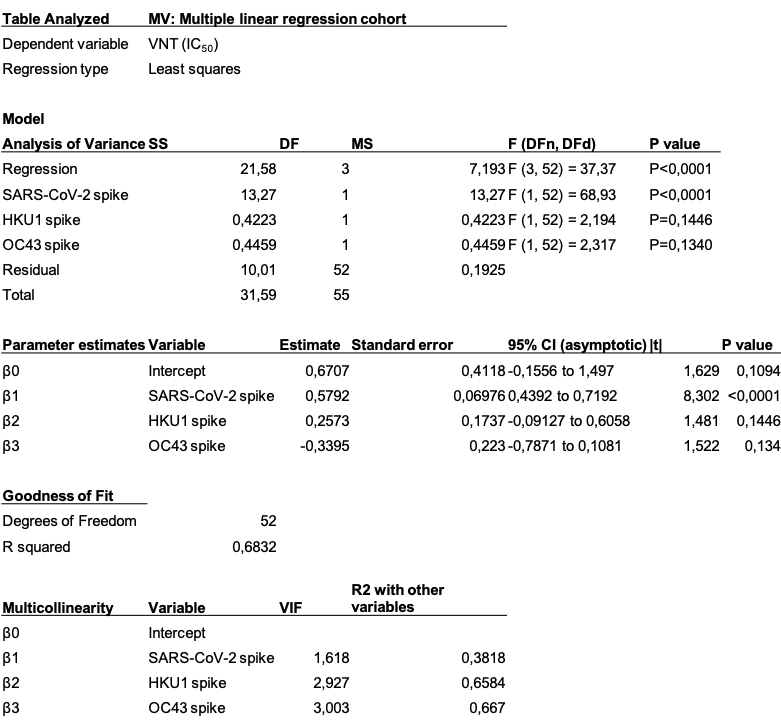
***

***Table S2. Tabular statistics of multiple linear regression analysis without interaction.***

***
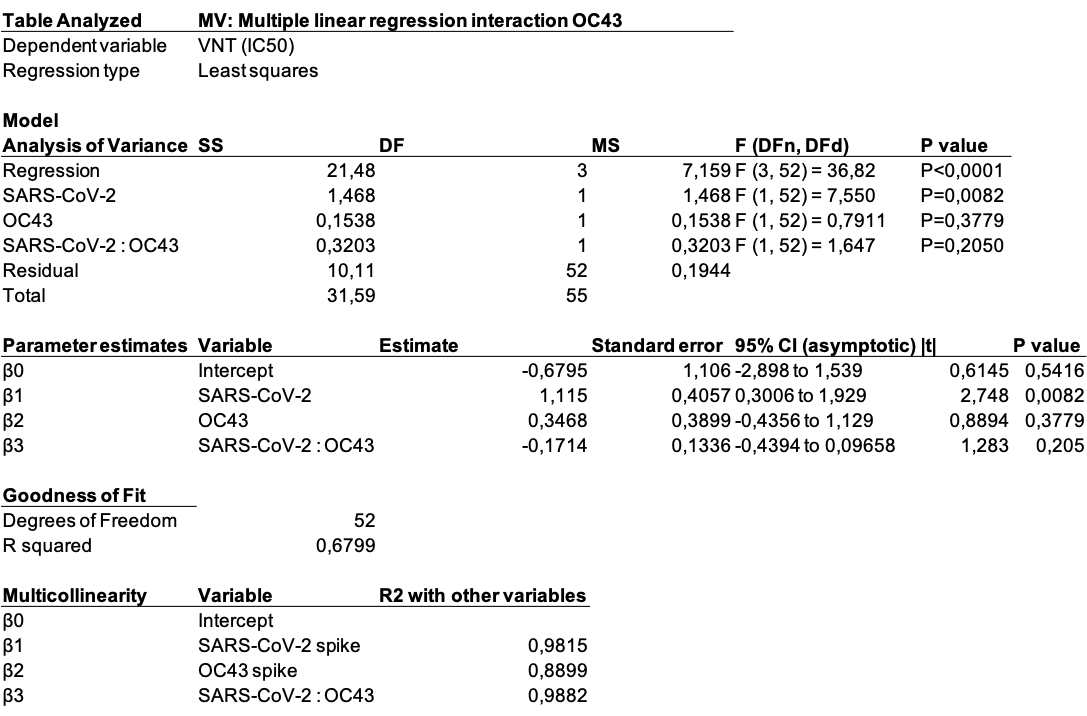
***

***Table S3 Tabular statistics of multiple linear regression analysis with interaction OC43 : SARS-CoV2.***

*
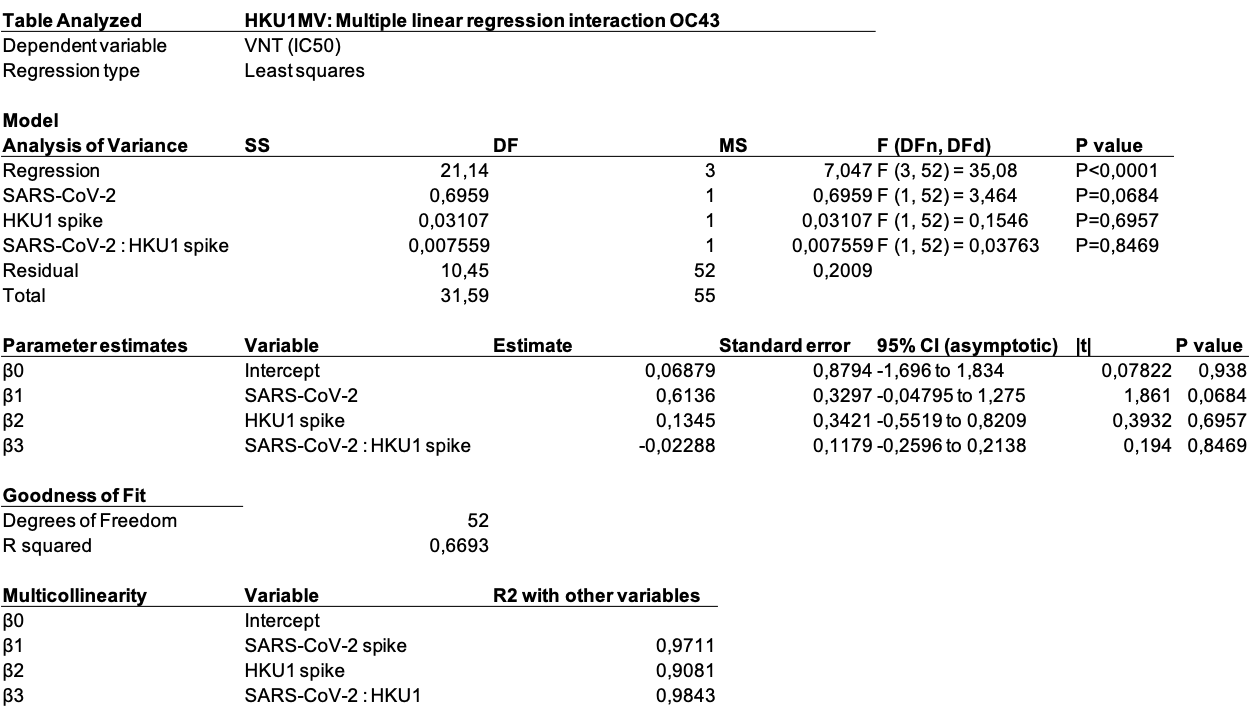
* ***Table S4 Tabular statistics of multiple linear regression analysis with interaction HKU1 : SARS-CoV2.***
